# Altered Phenotype of Circulating Dendritic Cells and Regulatory T Cells from Patients with Acute Myocarditis

**DOI:** 10.1155/2022/8873146

**Published:** 2022-02-28

**Authors:** Paola del Carmen Guerra-de-Blas, Daniela Cruz-González, Elena B. Martínez-Shio, Roberto González-Amaro, Héctor González-Pacheco, Esther Layseca-Espinosa, Carlos D. Escobedo-Uribe, Adriana E. Monsiváis-Urenda

**Affiliations:** ^1^The Mexican Emerging Infectious Diseases Clinical Research Network (LaRed), Ciudad de México, Mexico; ^2^Medicina Molecular Y Traslacional, Centro de Investigación en Ciencias de la Salud Y Biomedicina, Facultad de Medicina, Universidad Autónoma de San Luis Potosí, Mexico; ^3^Nutrition Deparment, Universidad de Matehuala, Mexico; ^4^Departamento de Urgencias Y Unidad Coronaria, Instituto Nacional de Cardiología “Ignacio Chávez”, Ciudad de Mexico, Mexico; ^5^Departamento de Cardiología, Facultad de Medicina, Universidad Autónoma de San Luis Potosí, Mexico

## Abstract

Dendritic cells (DCs) and regulatory T cells (Tregs) play an essential role in myocarditis. However, a particular DC phenotype in this disease has not been assessed. Herein, we aim to evaluate myeloid (mDCs) and plasmacytoid DC (pDC) phenotype, as well as Treg levels from myocarditis patients and healthy controls. Using multiparametric flow cytometry, we evaluated the levels of myeloid DCs (mDCs), plasmacytoid DCs (pDCs), and Tregs in peripheral blood from myocarditis patients (*n* = 16) and healthy volunteers (*n* = 16) and performed correlation analysis with clinical parameters through Sperman test. DCs from myocarditis patients showed a higher expression of costimulatory molecules while a diminished expression of the inhibitory receptors, ILT2 and ILT4. Even more, Treg cells from myocarditis patients displayed higher levels of FOXP3 compared to controls. Clinically, the increased levels of mDCs and their higher expression of costimulatory molecules correlate with a worse myocardial function, higher levels of acute phase reactants, and higher cardiac enzymes. This study shows an activating phenotype of circulating DCs from myocarditis patients. This proinflammatory status may contribute to the pathogenesis and immune deregulation in acute myocarditis.

## 1. Introduction

Myocarditis is an inflammatory disease of the myocardium with a wide range of clinical manifestations that are potentially devastating [1]. The global mortality burden of myocarditis is significant [2], with an estimated prevalence in 2015 of 1.56 million worldwide. However, the true incidence of myocarditis is unknown due to the low degree of suspicion and to the high possibility of misdiagnosis [3]. Myocarditis is related to SCD (sudden cardiac death) and dilated cardiomyopathy. Up to 42% of cases of SCD had a diagnosis of myocarditis, suggesting a significant epidemiological impact [4]. Although this disease may affect individuals of all ages, it is most frequent in young people [5].

Myocarditis can be associated with infectious, immunologic, or toxic conditions. The precise etiology remains undetermined in many patients [6]. Despite the causes, the pathogenic immune process in the heart leads to chronic inflammation, tissue remodeling, fibrosis, muscle fiber damage, systolic dysfunction, and finally, dilated cardiomyopathy (DCM) [7].

The diagnosis of acute myocarditis is based on the set of signs and symptoms, electrocardiographic abnormalities, elevated acute phase reactants, and specific serum biomarkers such as troponin [5]. Recently, specialized techniques such as magnetic resonance images have become part of the diagnostic algorithm of these patients due to its noninvasive diagnosis and good performance, with diagnostic accuracy up to 85% and 100% of negative predictive value [8].

It is known that different inflammatory cells, resident inflammatory cells, as well as circulating immune cells have an important role in the immunopathogenesis of myocarditis [7]. After acute myocardial viral infection, the immune cells are recruited to the infected heart. These include macrophages, dendritic cells, and T lymphocytes. The immune response can induce infected cardiomyocytes to die. However, surviving cells may give rise to persistent infection. Both situations may be responsible for acute or chronic myocarditis, respectively [9]. It has been postulated that acute viral myocarditis is characterized by an inflammatory milieu, which turns into an anti-inflammatory environment after several days. This second phase is defined by the production of IL-4, IL-5, and IL-10 by different cells, including Tregs [10].

The balance in the host immune response is a key determinant for a good prognosis of myocarditis patients. Activation of the immune response assures the containing of infection. However, it can also cause damage to the myocardium. The proper regulation of the magnitude of the immune response is critical to avoid excessive tissue damage, which in turn would lead to myocardial dysfunction. In this regard, it is clear that both innate and adaptive immune responses have a role in the pathogenesis of myocarditis [11].

Dendritic cells (DCs) are a subpopulation of leukocytes specialized in the capture and process of antigens and its presentation to T lymphocytes. They are key elements of the innate and adaptive immunity. There are two major subsets of DCs, myeloid or classic DCs (mDCs) and plasmacytoid DC (pDCs). Human blood DCs are broadly defined as leukocytes that are HLA-DR positive and lack the expression of markers specific for T cell, B cell, NK cell, monocyte, and granulocyte lineages. They can be subdivided into two major subsets of DCs, myeloid or classic DCs (mDCs), and CD11c+, which can also express BDCA1, and CD11c- plasmacytoid DC (pDCs) that also express BDCA4+. BDCA-4 is also expressed on monocyte-derived DCs [12–14]. Immature mDCs show a great phagocytic activity and a poor immunogenic effect, while mature mDCs exhibit an enhanced capability to synthesize proinflammatory cytokines as well as a very good activity as antigen-presenting cells. pDCs are able to synthesize high levels of type I interferon, mainly IFN-*α*, in response to viral and other stimuli [15]. Despite its well-known ability to trigger the adaptive immune response, DCs have been recognized to play an important role in the maintenance of immune tolerance [16–18].

Regulatory T cells (Tregs) are a subpopulation of lymphocytes responsible for suppressing and maintaining self-tolerance. Tregs ensure a controlled immune response upon pathogen encounter and thereby prevent immune pathology. Conversely, excessive suppression by Tregs can hamper pathogen clearance and promote chronic infection [18, 19]. It has been described that DCs and Treg are closely linked. In peripheral tissues, the maintenance of Tregs depends on the costimulation signals provided by DCs. Specifically, the interactions of CD80 and CD86 on APCs with the CD28 receptor on T cells are responsible for Tregs maintenance [19]. The tolerogenic function of DCs relies on the inhibitory function of several receptors. Immunoglobulin-like transcript (ILT)-2 and -4 are involved in the tolerogenic ability of DCs by inhibiting activating functions of DCs [20]. Interestingly, it has been observed that early activation of Tregs may be associated with the exacerbation of viral myocarditis. In counterpart, attenuation of acute cardiac inflammation by Tregs seems to prevent the progression of myocarditis to DCM in humans [21]. It has been suggested that antigen-presenting cells, like macrophages and DC, are responsible for the production of inflammatory cytokines and chemokines during the initial phases of viral myocarditis [22]. Adoptive transfer of DCs loaded with cardiac antigens can induce the infiltration of CD4+ T cells into cardiac tissue and the development of experimental autoimmune myocarditis (EAM) [23] indicating that DCs are sufficient for inducing the disease. The activation of DCs by the proinflammatory cytokines interleukin 1 (IL 1) and granulocyte-macrophage colony-stimulating factor (GM-CSF) was found to be crucial for efficient autoreactive T cell responses and EAM induction. In vivo, inhibition of the costimulatory molecule CD80 decreased myocardial inflammation in a murine model [24, 25]. Another study described the contribution of the RNA-sensing receptor TLR7 to EAM severity, suggesting that TLR7 expressing DCs (such as pDCs) have a role in driving the disease [26].

Although there is evidence of DC involvement in myocarditis pathogenesis, little is known about their role in the severity of myocarditis. Furthermore, it has not been evaluated the phenotype, including costimulatory molecules and inhibitory receptor expression, of DCs from myocarditis patients. Herein, the aim of this study is to assess the levels and phenotype of circulating DC populations, mDCs and pDCs, as well as Tregs levels, from patients with nonautoimmune acute myocarditis and evaluate their correlation with inflammatory cytokines and the severity of the disease.

## 2. Materials and Methods

### 2.1. Patients

Sixteen patients with a diagnosis of acute myocarditis were included in the study. Age was between 22 and 45 years (mean age 34.1 years). Fifteen healthy controls were also included in this study. Medical record data were systematically extracted for epidemiologic factors (age, gender, comorbidities, duration of cardiac symptomatology, left ventricular ejection fraction by echocardiography, initial troponin, and initial creatine kinase MB). Twelve of the patients had a history of infection at the time of cardiac disease onset. A complete blood count marked by leucocytosis, raised C-reactive protein, and history of symptoms consistent with upper airway infection and/or gastroenteritis before presentation supported the suspicion of infection. In addition, to exclude autoimmunity, antinuclear antibodies were measured, and one patient was positive. All clinical and demographic data are shown in Table 1. A blood sample was obtained from patients within 12 h following admission to the Cardiology Department of the National Institute of Cardiology “Ignacio Chavez”. All control samples were obtained from healthy volunteers.

A cardiologist from the National Institute of Cardiology “Ignacio Chavez” made the myocarditis diagnosis. The clinical diagnosis of myocarditis was based upon the current recommendations given by the European Society of Cardiology Working Group 5. The criteria considered were as follows:

- Clinical presentation: symptoms related to systemic inflammatory response and manifestations of cardiovascular disease such as dyspnea, palpitations, and chest pain
- ECG features: sinus tachycardia, abnormal PR, ST and T wave, and AV block
- Elevation of biomarkers: Increased serum levels of troponin T not explained by other causes
- Echocardiographic: decreased systolic ventricular function and/or alterations of ventricular segmental mobility
- Magnetic Resonance Imaging (MRI): presence of 2 of the following (1) Regional or global myocardial signal intensity increase in T2-weighted, myocardial edema images characterized by myocardial edema in the T2 hyperintensity, (2) hyperemia and impaired capillary characterized by increased global myocardial early gadolinium enhancement ratio between myocardium and skeletal muscle in gadolinium-enhanced T1-weighted images, and (3) necrosis and/or fibrosis characterized by late gadolinium enhancement

In all cases, informed written consent was obtained. This study was approved by the local Ethics Committee. This work was carried out in accordance with The Code of Ethics of the World Medical Association for experiments involving humans.

### 2.2. Cell Isolation

Peripheral blood mononuclear cells (PBMCs) were isolated using Ficoll Hypaque (Sigma Chemical Co., St. Louis, MO) centrifugation. Then, PBMC were labelled and analyzed using flow cytometry.

### 2.3. Antibodies

The following monoclonal antibodies (mAb) were used: FITC antihuman Lineage Cocktail (Clone M*φ*P9, NCAM16.2, 3G8 SK7, and L27 SJ25C1), APC-Cy7 anti-HLA-DR (L243), and PerCP-Cy5.5 anti-CD11c (B-ly6) (BD Biosciences, San Jose, CA); anti-BDCA1(AD5-8E7) and anti-BDCA4 tagged with APC (AD5-17F6) (Miltenyi Biotech), PE labeled anti-ILT4 (42D1), anti-ILT2 (GHI/75), anti-CD40 (5C3), anti-CD80 (2D10), or anti-CD86 (IT2.2) (BioLegend, San Diego, CA). For Tregs lymphocytes, anti-CD25 labeled with APC (M-A251) and PE anti-FOXP3 (259D/C7) (Becton-Dickinson, San Jose, CA) antibodies were used.

### 2.4. Flow Cytometry Analysis

PBMC labelling was based on a procedure previously published by our team [27], PBMC were labelled with 5 *μ*l of a FITC antihuman lineage antibody cocktail (anti-Lin) (CD3, CD14, CD16, CD19, CD20, and CD56), 7 *μ*l of APC-Cy7 tagged antihuman-HLA-DR, 5 *μ*l of PerCp-Cy5.5 labelled antihuman CD11c, 7 *μ*l of APC labelled antihuman BDCA1 or antihuman BDCA4 and 5 *μ*l of antihuman ILT4 PE, for 20 min at 4°C. Then, cells were washed, fixed with 1% PFA, and analysed in a FACSAria II cytometer (BD Biosciences). PBMC were also labelled with 3 *μ*l of FITC antihuman-CD80, PerCP-Cy5 antihuman-CD86, and PE antihuman-CD40. In all assays, Fc receptors were blocked with 10% human AB serum. Flow cytometry data were analysed using the FACSDiva (BD Biosciences) and FlowJo software (Tree Star Inc., Ashland, OR). For gates settings, we used the FMO (fluorescence minus one) strategy. In brief, FMO controls leave out one reagent at a time.

For the flow cytometry analysis of T regulatory cells, PBMC were washed and double stained with a FITC anti-CD4 and APC anti-CD25 mAb (Becton-Dickinson, San Jose, CA). For intracellular staining, cells were washed, fixed, and permeabilized with the Foxp3 Fix/Perm kit (eBioscience) for 30 minutes. Then mononuclear cells were stained with anti-Foxp3. In all cases, at least 1 × 106 events were acquired, and gates also were established using the fluorescence minus one strategy in a FACSAria II flow cytometer (Becton Dickinson). Data were analysed using the FACSDiva and FlowJo software.

### 2.5. Cytokine Production

Cytokine levels were determined in serum from patients and controls using the cytokine bead array (CBA) kit for inflammatory cytokines (BD Biosciences). Cytokine levels were quantified by following manufacturer instructions and then analysed using FACS Canto II (BD Biosciences).

### 2.6. Statistical Analysis

Data were analysed with the GraphPad Prism, 5.01 software. Flow cytometry data were evaluated using the Mann–Whitney *U* test. When indicated, the Kruskal-Wallis test was also performed. Analysis post hoc was made using the Dunnet post-test.

The analysis of correlation between variables was based on Spearman's rank test. *p* < 0.01 was considered statistically significant.

## 3. Results

### 3.1. Clinical Characteristics of Myocarditis Patients

We studied sixteen male myocarditis patients. All clinical and demographic characteristics are shown in Table 1. Twelve patients had a history of previous infection. Although it was not possible to assess the precise etiology of myocarditis, we assumed on the basis of the clinical presentation and epidemiologic data, viral infection was the most probable cause of myocarditis. The following data was obtained at the moment of diagnosis: The mean of days with infection symptoms was 5.6 ± 3.9; the mean days with cardiac symptomatology was 3 ± 2.9. The blood test showed that patients had a mean white blood cell count 12585 ± 3248, and the mean C-reactive protein was 37.96 ± 35.2 mg/L, troponin mean levels were 27.74 ± 41.48 ng/ml, and the mean levels of creatine kinase fraction MB were 106.3 ± 214.37. Thirteen patients have a left ventricular ejection fraction (LVEF) higher than 50, and three have a left ventricular ejection fraction less than 50. Fourteen patients had ECG abnormalities (ST segment elevation), and two patients showed arrhythmia (Table 1). Controls were sex-matched and age-matched, with a mean age of 34.5 ± 8.9.

### 3.2. Myocarditis Patients Have Increased Levels of Myeloid and Plasmacytoid Dendritic Cells

**Figure 1:**
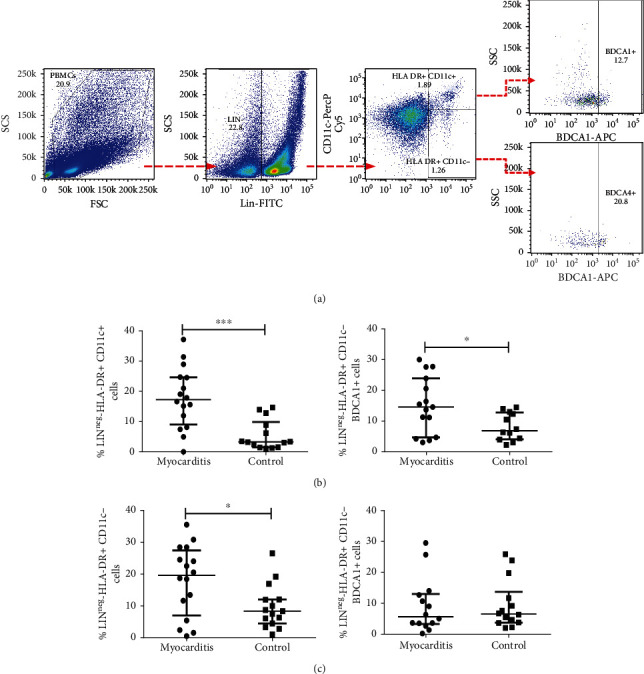
Percentages of plasmacytoid dendritic cells and myeloid dendritic cells from myocarditis patients and controls. PBMC from myocarditis and control subjects were immunostained and analyzed by five-color flow cytometry. (a) Gating tree from negative lineage marker (LINneg), HLADR + CD11c+, and BDCA1+ (myeloid dendritic cells-mDCs), and from HLADR + CD11c− and BDCA4+ (plasmacytoid dendritic cells-pDCs). (b) Percentage of myeloid DCs (LINnegHLA − DR + CD11c+) and BDCA1+ from myocarditis and healthy volunteers. (c) Percentage of plasmacytoid DCs (LINnegHLA − DR + CD11c−) and BDCA4+ from myocarditis patients and controls. ^∗^*p* < 0.05, ^∗∗^*p* < 0.0.

In order to assess the levels of plasmacytoid and myeloid dendritic cells, we performed multiparametric flow cytometry employing the following strategy of gating: from FSC and SSC plot lineage (LIN) negative population was selected; myeloid dendritic cells were defined as HLA − DR + CD11c+, while plasmacytoid dendritic cells were considered as HLA − DR + CD11c− (Figure 1(a)). For further phenotyping, we assessed the expression of blood dendritic cell antigen-1 (BDCA1+) in myeloid dendritic cells (HLA − DR + CD11c+) and blood dendritic cell antigen-4 (BDCA − 4) in plasmacytoid dendritic cells (HLA − DR + CD11c−), as shown in Figure 1(a). We found that myocarditis patients displayed increased percentages of myeloid and plasmacytoid DCs (*p* < 0.05, Figure 1(b)) compared with healthy controls. It was noticeable that when BDCA markers were included in the analysis, only BDCA1 + DCs were increased.

### 3.3. Expression of Costimulatory Molecules in Plasmacytoid Dendritic Cells and Myeloid Dendritic Cells

**Figure 2:**
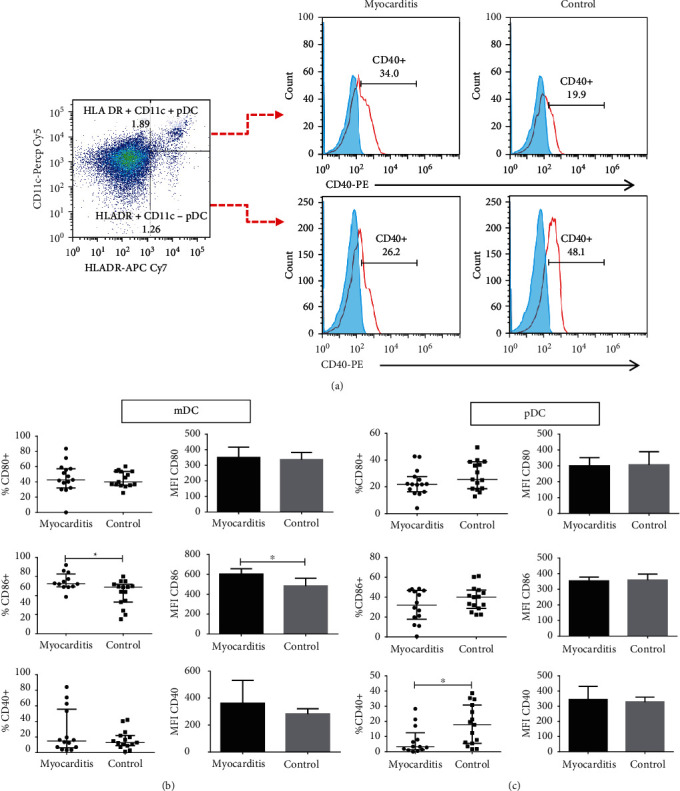
Percentages of costimulatory molecules in plasmacytoid and myeloid dendritic cells. PBMC from myocarditis and controls were immunostained, and cells were analyzed using flow cytometry. (a) Gating tree from lineage negative (LINneg), HLADR + CD11c + DCs (mDCs) additionally labeled for CD40+, CD80+ or CD86+, and (LINneg), HLADR+ CD11c− (pDCs) additionally labeled for CD40+, CD80+, or CD86+. (b) Percentages (left) and MFI (right) of costimulatory molecules in myeloid DCs. (c) Percentages (left) and MFI (right) of costimulatory molecules in plasmacytoid dendritic cells. ^∗^*p* < 0.05, ^∗∗^*p* < 0.01.

Since activation status of DCs conditions their ability to prime T cells, we decided to evaluate the expression of the costimulatory molecules CD80, CD86, and CD40, in both myeloid and plasmacytoid DCs, as shown in Figure 2(a). Interestingly, we found that myocarditis patients showed higher percentages of CD86 positive myeloid DCs compared with healthy controls (*p* = 0.01, Figure 2(b)). This was in accordance with their levels of surface expression, measured as mean fluorescence intensity (MFI) (*p* = 0.01, Figure 2(b)). CD80 and CD40 costimulatory molecules showed no differences between controls and patients. Furthermore, plasmacytoid DCs from myocarditis patients showed lower percentages of CD40 positive cells (*p* = 0.01, Figure 2(c)) compared with healthy controls. Costimulatory molecules CD86 and CD80 did not show significant changes when compared with healthy volunteers.

### 3.4. ILT2 & ILT4 Expression in Circulating Dendritic Cells from Patients with Myocarditis

**Figure 3:**
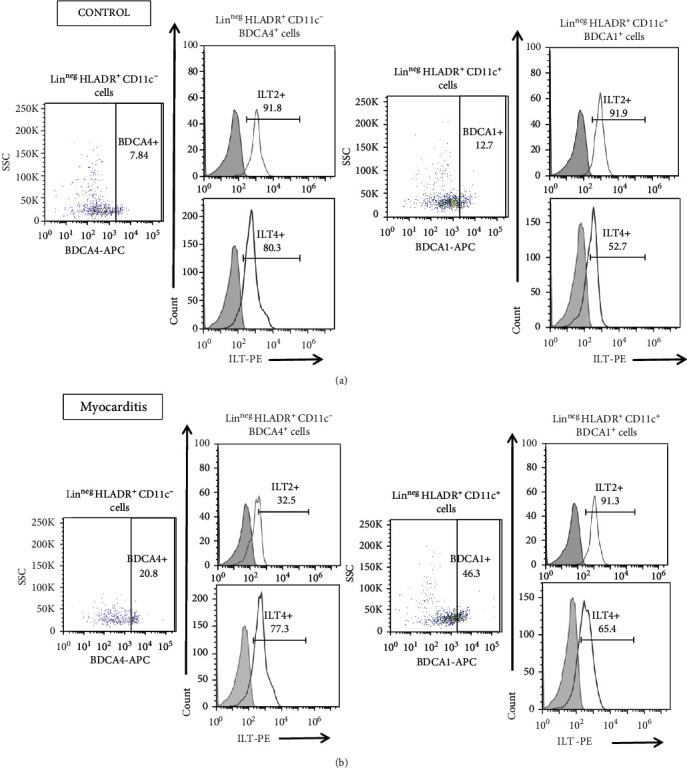
Expression of ILT2 and ILT4 in DCs from myocarditis and control subjects. Multiparametric flow cytometry gating tree for lineage negative (LINneg), HLA − DR + CD11c+, BDCA1+ ILT2+ or ILT4+ cells (mDCs) and (LINneg), HLA − DR + CD11c−, BDCA4+, and ILT2+ or ILT4+ (pDCs). Gating tree for the analysis of the expression of ILT2 and ILT4 in myeloid dendritic cells and plasmacytoid dendritic cells in PBMC from a control subject (a) and (b) a myocarditis patient. Myeloid dendritic cells showed similar percentages of ILT2 and ILT4 positive DCs compared with healthy volunteers (Figures 4(b) and 4(d)). Interestingly, myeloid DCs from myocarditis patients displayed higher surface expression (MFI) of ILT4 compared to controls (*p* = 0.03, Figure 4(d)).

It is proposed that inhibitory receptors expressed in antigen-presenting cells (APCs) provide them with tolerogenic functions. The expression of the inhibitory receptors ILT2 and ILT4 in DCs may contribute to regulating the immune-inflammatory response. We decided to evaluate ILT2 and ILT4 expression on DCs by multiparametric flow cytometry. Using a similar labeling and gating strategy published by Monsivaís-Urenda [28], we analyzed the percentage of ILT2 and ILT4 positive cells in myeloid dendritic cells defined as LINnegHLA − DR + CD11c + BDCA1+ and plasmacytoid dendritic cells defined as LINnegHLA − DR + CD11c − BDCA4+ in circulating dendritic cells from myocarditis patients (Figure 3(a)) and healthy controls (Figure 3(b)).

**Figure 4:**
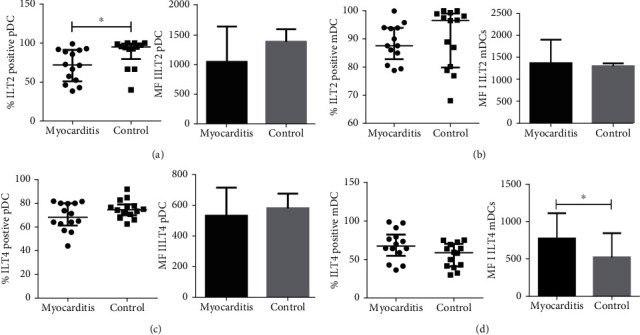
Expression of ILT2 and ILT4 in DCs from myocarditis and control subjects. Percentage of ILT2 (a) and ILT4 (c) in plasmacytoid DCs: LINnegHLADR+CD11c−BDCA4+ (right) and ILT2 and ILT4 expression on pDCs (MFI) (right). Percentage of ILT2 (b) and ILT4 (d) in myeloid DCs: LINneg, HLADR+CD11c + BDCA1+ (left) and expression of ILT2 and ILT4 mean fluorescence intensity on mDCs (MFI) (right). Data are shown as median and interquartile range. ^∗^*p* < 0.05, ^∗∗^*p* < 0.01.

Remarkably, myocarditis patients showed lower percentages of ILT2 positive plasmacytoid dendritic cells compared to controls. (*p* = 0.01, Figure 4(a)). Furthermore, the surface expression (MFI) of ILT2 shows a tendency to be lower in myocarditis patients compared to controls (Figure 4(a)). However, the ILT4 level of expression did not show differences between both groups (Figure 4(c)).

### 3.5. Myocarditis Patients Show Higher Levels of FOXP3 Expression in Regulatory T Cells

**Figure 5:**
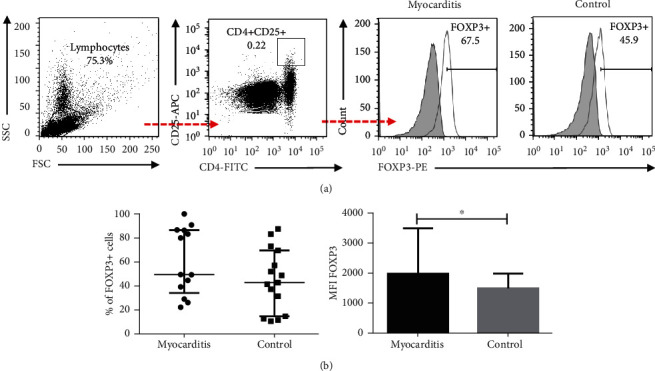
Regulatory T cells from myocarditis patients and controls. PBMC from myocarditis and control subjects were immunostained, and then cells were analyzed by five-color flow cytometry. (a) Gating tree for CD4 + CD25 + FOXP3+ cells. (b) Percentage of FOXP3+ cells in Treg cells from myocarditis patients and controls. (c) Median fluorescence intensity (MFI) of FOXP3+ cells in CD4 + CD25+ lymphocytes from myocarditis patients and controls. ^∗^*p* < 0.05, ^∗∗^*p* < 0.01.

Myocarditis prognosis relies on the correct balance between inflammation and immune regulation. Therefore, regulatory T cells may play an important role in counterbalancing immune activation. We decided to assess the levels of regulatory T cells, defined as CD4 + CD25 + FOXP3+ lymphocytes, from myocarditis patients and controls. The gating strategy is defined in Figure 5(a). We found that myocarditis patients showed similar levels of Tregs compared to the controls (*p* > 0.05, Figure 5(b)). However, when we analyzed the surface expression (MFI) of FOXP3 in these cells, we observed that myocarditis Treg lymphocytes expressed higher levels of FOXP3 (*p* = 0.04, Figure 5(b)).

### 3.6. Myocarditis Patients Showed Higher Levels of IL-6

**Figure 6:**
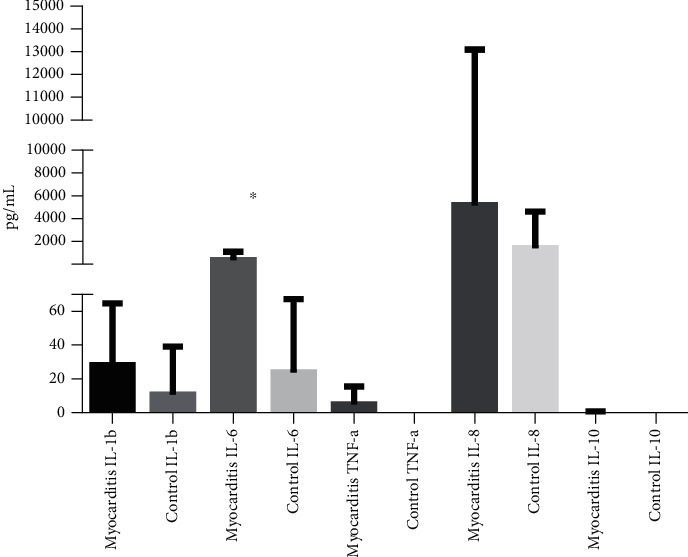
Cytokine levels in the plasma of patients with myocarditis and control subjects. The concentrations of the indicated cytokines were determined using flow cytometry in plasma from myocarditis patients and healthy subjects. ^∗^*p* < 0.05, ^∗∗^*p* < 0.01.

It is known that proinflammatory cytokines are important prognostic clues in myocarditis. We measured the levels of the proinflammatory cytokines IL-1*β*, IL-6, IL-8, and the regulatory cytokine IL-10 in myocarditis and control serum. IL-8 levels were highly heterogeneous in the myocarditis group, but we could observe a tendency to be higher compared to controls. However, IL-6 was significantly higher in myocarditis serum compared to controls (Figure 6). TNF-*α* and IL-1*β* showed no differences between groups. IL-10 and could not be detected in the serum from myocarditis patients nor controls.

### 3.7. Association between Clinical Characteristics in Myocarditis Patients and the Proportion and the Phenotype of Dendritic and Regulatory T Cells

To our knowledge, there are no previous reports about the associations between phenotypic characteristics of DCs, regulatory T cells, and clinical parameters such as left ventricular ejection fraction (LVEF), creatine kinase fraction MB (CK-MB), troponin-I, C-reactive protein, duration of cardiovascular symptoms (cardiovascular disease evolution time, CET), and with the duration of infectious symptoms (infection evolution time, IET).

Interestingly, we found that there was a negative correlation between BDCA1 positive mDCs and LVEF (*r* = −0.53, *p* = 0.04). Even more, the surface expression (MFI) of the costimulatory molecule CD40 in mDCs had a negative correlation with LVEF (*r* = −0.06, *p* = 0.01). Furthermore, the percentage of CD86 positive mDCs displayed a positive correlation with the levels of creatine kinase fraction MB (*r* = 0.6, *p* = 0.02). There was no correlation between any clinical parameters and the other costimulatory molecules expressed by mDCs (Table 2).

On the other hand, BDCA4+ pDCs correlated positively with the levels of creatine kinase fraction MB (*r* = 0.62, *p* = 0.03) and with the levels of troponin I (*r* = 0.66, *p* = 0.04). Costimulatory molecules CD80, CD86, and CD40 expressed by pDCs did show an association with different clinical parameters. We found that the percentage of CD86 positive cells correlated positively with the levels of troponin-I (*r* = 0.6, *p* = 0.03). Furthermore, the surface expression of this molecule had a positive correlation with the levels of troponin-I (*p* = 0.0001). Interestingly, the percentage of CD86 positive cells showed a negative correlation with the evolution time of the disease (*r* = −0.62, *p* = 0.009). The surface expression of the costimulatory molecule CD40 in pDCs had a positive correlation with the levels of C-reactive protein (*r* = 0.61, *p* = 0.02) (Table 3).

It is important to highlight that FOXP3 expression by Treg cells was positively associated with the evolution time of the disease (*r* = 0.69, *p* = 0.01) (Table 4).

## 4. Discussion

DCs are well recognized as the most efficient antigen-presenting cells, and they have emerged as key participants in the initiation of adaptive immune responses. However, DCs are critical for immune regulation and maintenance of peripheral tolerance [29].

DCs play an important role in the immunopathogenesis of different autoimmune and inflammatory diseases. They contribute to the development of myocarditis. The participation of DCs in different cardiomyopathies and their effects on cardiac function has been described. In the onset of acute myocardial infarction, the number of mDCs and pDCs decreases while their levels restore after the acute event. Even more, DC numbers correlate positively with the concentration of CK-MB and brain natriuretic peptide (BNP) [30]. Nevertheless, to our knowledge, not only the percentages but also the phenotype of DC in acute myocarditis have not been assessed.

It is important to mention that different experimental approaches for defining myeloid (mDC) and plasmacytoid (pDC) populations may produce different results. According to their hematopoietic origin, DCs can be divided into two major subsets, mDCs and pDCs. They can be differentiated by their expression of the myeloid antigen CD11c [31]. Also, it has been described that circulating mDCs express BDCA1; BDCA2 and BDCA4 are expressed by pDCs [12]. However, it has been reported that not all pDCs are positive for the BDCA4 antigen [32]. We found higher levels of circulating mDCs BDCA1 positive cells in myocarditis patients compared to healthy controls. We also found significantly higher levels of HLA − DR + CD11c− DCs, which correspond to the plasmacytoid population; however, when BDCA4 was included in the analysis, no differences were found. Therefore, our results indicate that among the pDC population, a specific population of pDCs may be involved in the acute phase of myocarditis. This result highlights the importance of considering not only the BDCA markers to define DC phenotype. It would have been of interest to evaluate BDCA2 since it has not been reported that BDCA4 and BDCA2 may represent two different pDC populations. The increment in circulating DCs may contribute to the recruitment of leukocytes to the heart observed in myocarditis as a result of chemokine production by other immune populations. In line with this, it has been demonstrated that the number of cardiac dendritic cells is increased significantly during the acute and subacute phases of myocarditis [33, 34].

Myocarditis is a common cause of dilated cardiomyopathy [35], which, in turn, is a consequence of chronic myocardium inflammation [36–38]. The expression of costimulatory molecules by DCs determines T cell activation. Interaction of CD28 and CD40L on T cells with CD80, CD86, and CD40 on APC, respectively, is crucial for proper activation and proliferation of T cells [39–41]. Accordingly, we decided to evaluate the expression of costimulatory molecules in DCs. The higher expression of CD86 in DCs from myocarditis patients suggests that in this condition, DCs present a higher immunogenic status increasing their ability to prime T cells. Furthermore, it has been reported that in vivo administration of anti-CD80/CD86 mAb markedly decreased myocardial inflammation [24]. Our results provide one possible mechanistic explanation to this observation and support the possibility of using mAb to reduce myocardial inflammation and progression to DCM. In this regard, Athanassopoulos et al. evaluated DCs in peripheral blood from DCM patients, and they found that the levels of mDCs and pDCs are increased significantly in these patients compared to other cardiac diseases (coronary artery disease, hypertrophic cardiomyopathy, nonischemic dilated cardiomyopathy, severe valvular disease, etc.) and compared with healthy controls. They also found that the maturation marker CD83 and the homing receptor to lymphoid CCR7 were increased on mDCs from DCM patients [41]. It is important to point out that the patients included in our study were in the acute phase of the disease, and we did not include any DCM patients.

Cytokines are important players triggering and determining the overcome of myocarditis. Even more, it is well known that mature DCs are great producers of cytokines. Our results showed increased levels of IL-6 and IL-8 in serum from myocarditis patients. It has been reported that the proinflammatory cytokines IL-1*β*, IL-6, and TNF-*α* are increased in acute myocarditis [42–44]. There is consistency between the increased expression of costimulatory molecules in myocarditis DCs and the higher levels of IL-6 and IL-8 observed. In this regard, it is very interesting that recent reports highlight the importance of IL-6 levels associated with a severe presentation of viral diseases. It has been shown that SARS-CoV-2 severe cases are associated with higher levels of circulating IL-6, IL-2R, TNF-*α*, and IL-10, suggesting cytokines might be associated with disease severity [45]. In our study, we cannot dismiss the different clinical scenarios displayed by our patients, including the duration of infection, symptomatology, and CRP (C-reactive protein) levels.

The undetectable amounts of IL-10 were evident, however more research is needed to assses the possible production of IL-10 by different immune cell populations infiltrating myocardial tissue. In this regard, we analyzed the expression of the inhibitory receptors ILT2 and ILT4. It is known that the expression of these receptors induces DC tolerance [34, 35]. Consistently with the low levels of IL-10, we did not find differences in the expression of ILT4 in mDCs or pDCs. However, ILT2 expression on pDCs was lower in the myocarditis patients, which is in accordance with the activating phenotype found.

It has been demonstrated in a well-established viral model of myocarditis that it can occur in three phases: infection, autoimmune reaction, and dilated cardiomyopathy, each of them characterized by different symptomatic presentations: malaise, inflammation, and heart failure. Phase 1 is represented by the production of inflammatory cytokines. Phase 2 is induced by the presentation of antigen by DCs to T cells. Inflammation can be regulated or go on chronically as an autoimmune reaction [46]. The latter results are important since most of our patients were negative for antinuclear antibodies (ANA), suggesting they were in phase 1 at the moment of the study.

T regulatory cells are responsible for maintaining immune homeostasis and tolerance. They can suppress T cell activation directly by secreting immune-suppressive cytokines like IL-10 or TGF-*β* or indirectly through modulation of antigen cell presenting cells (DCs) [47]. Tregs can inhibit the maturation of DCs, decreasing the expression of costimulatory molecules CD80, CD86, and CD40 [48]. The role of Tregs in myocarditis is yet controversial; it has been reported that they could have a beneficial role in preventing the initiation of autoimmunity, ameliorating disease progression, or ameliorating tissue damage [49, 50]. Huber demonstrated in a murine model of coxsackievirus B3-induced myocarditis that higher levels of Treg cells resulted in lower inflammation of the myocardium [51]. Conversely, Zheng et al. found that high FOXP3 expression resulted in aggravated myocardial damage [52]. In line with these results, we found higher levels of FOXP3 expression in the myocarditis patients. Furthermore, FOXP3 expression positively correlates with the disease evolution time. Whether if the increment of FOXP3 expression on Tregs is a result of inflammatory cytokines or it is a counterbalance for the inflammatory state needs further investigation.

A limitation of this study is the small sample size. Despite this limitation, our results show novel and statistically significant findings about DC levels and phenotype from myocarditis patients. Furthermore, our results highlight the contribution of DCs and their proinflammatory status to the pathogenesis and immune deregulation in acute myocarditis, and even more, it opens new lines for further research in larger cohorts.

An important contribution of this study is our preliminary findings regarding the correlation between immune parameters and myocardial function. As expected, higher levels of acute-phase reactants and higher cardiac enzymes correlate with a worse myocardial function. It was interesting that, in an overall view, higher levels of costimulatory molecules correlate with a worse myocardial function. However, it was noticeable that higher levels of pDCs were correlated with less time of disease. We hypothesize that activation of DCs induces the production of inflammatory cytokines, such as IL-6, which in turn induce acute phase reactants (CRP). The inflammatory milieu may favor myocardial damage, but at the same time, it may also provide a mechanism to eliminate virus infection leading to a faster recovery. In contrast, Tregs may interfere in the resolution of infection due to the early phases of the disease approached in this study.

## 5. Conclusions

In summary, these results show that myocarditis patients display higher levels of circulating DCs with an activating phenotype. This proinflammatory status, together with the impaired expression of FOXP3 in Tregs, may contribute to the pathogenesis and immune deregulation in acute myocarditis. The above joined with higher levels of IL-6 may contribute to damage to cardiomyocytes and attenuated cardiac function. The latter suggests a possible benefit of IL-6-directed therapy in myocarditis patients. Whether if the increment of FOXP3 expression on Tregs is a result of inflammatory cytokines or it is a counterbalance for the inflammatory state needs further investigation. Our results provide new knowledge about the possible contribution of DCs to myocarditis pathogenesis. Even more, our preliminary results show possible associations between the clinical severity of myocarditis and DC levels and phenotype, which contribute to the identification of future diagnostic, prognostic, or therapeutic targets in this complex pathology.

## Figures and Tables

**Table 1:** Clinical characteristics of patients with myocarditis.

	Myocarditis	Controls
Sex (M/F)	16/0	15/0
Age (years, mean ± SD)	34.1 ± 9.3	34.5 ± 8.9
History of infection	14	0
Duration of infectious symptomatology (days)	5.6 ± 3.9	
Duration of cardiac symptomatology (days)	3 ± 2.9	
Positive antinuclear antibodies	1	
White blood cells count	12585 ± 3248	
C-reactive protein (mg/L)	37.96 ± 35.2	
Ejection fraction (%)		
>50	13	
<50	3	
ECG abnormalities	14	
Arrhythmia	2	
Troponin (ng/mL)	27.74 ± 41.48	
CK-MB	106.3 ± 214.37	

ECG: electrocardiogram); CK-MB: creatine kinase MB.

**Table 2:** Correlation between phenotypic characteristics of myeloid dendritic cells (mDCs) with different clinical parameters.

Myeloid dendritic cells									
Clinical parameter		% LINnegHLA − DR + CD11c+	% LINnegHLA − DR + CD11c + BDCA1+	% CD80	CD80 (MFI)	% CD86	CD86 (MFI)	% CD40	CD40 (MFI)
LVEF (%)	r	-0.043	-0.532	0.111	−0.153	0.269	0.244	−0.013	−*0.063*
	*p*	0.844	^∗^ *0.0403*	0.691	0.584	0.349	0.396	0.954	^∗^ *0.0177*
CK-MB (ng/mL)	r	0.207	−0.007	0.182	−0.154	*0.600*	0.255	0.024	−0.015
	*p*	0.458	0.985	0.515	0.617	^∗^ *0.0261*	0.376	0.940	0.964
Troponin-I (ng/mL)	r	−0.156	0.038	0.005	−0.364	0.301	0.095	0.112	−0.133
	*p*	0.594	0.906	0.993	0.246	0.342	0.770	0.733	0.683
CRP (mg/L)	r	0.208	0.145	0.118	0.125	−0.528	0.479	0.033	0.262
	*p*	0.438	0.605	0.676	0.671	0.067	0.098	0.916	0.366
CET (days)	r	0.225	−0.107	0.265	−0.241	−0.276	−0.058	0.086	−0.165
	*p*	0.398	0.566	0.246	0.283	0.247	0.703	0.770	0.452
IET (days)	r	0.217	−0.163	−0.006	0.520	−0.504	−0.028	0.000	0.397
	p	0.477	0.583	0.964	0.105	0.088	0.903	0.974	0.203

LVEF: left ventricular ejection fraction; CK-MB: creatine kinase; CET: cardiovascular disease evolution time; CRP: C-reactive protein; IET: infection evolution time; r: correlation coefficient; p: probability value ^∗^*p* < 0.05, ^∗∗^*p* < 0.01, and ^∗∗∗^*p* < 0.001.

**Table 3:** Correlation between phenotypic characteristics of myeloid dendritic cells (mDCs) with different clinical parameters.

Plasmacytoid dendritic cells									
Clinical parameter		% LINnegHLA − DR + CD11c−	% LINneg HLA − DR + CD11c− BDCA4+	% CD80	CD80 (MFI)	%CD86	CD86 (MFI)	%CD40	CD40 (MFI)
LVEF (%)	r	0.424	0.303	−0.052	−0.273	−0.188	−0.284	0.065	−0.348
	*p*	0.102	0.290	0.834	0.304	0.508	0.310	0.828	0.235
CK-MB (ng/mL)	r	0.159	*0.629*	0.314	0.225	0.165	0.392	−0.240	0.005
	*p*	0.554	^∗^ *0.0317*	0.254	0.416	0.573	0.166	0.409	0.993
Troponin-I (ng/mL)	r	−0.066	0.661	0.424	0.084	*0.608*	*1.000*	−0.071	0.273
	*p*	0.817	^∗^ *0.0438*	0.132	0.776	^∗^0.0399	^∗∗∗^ *0.0001*	0.821	0.391
CRP (mg/L)	r	0.060	−0.266	0.214	0.467	0.213	0.130	0.152	*0.615*
	*p*	0.824	0.357	0.442	0.081	0.464	0.656	0.605	^∗^ *0.0284*
CET (days)	r	−0.260	−0.394	−0.407	−0.141	−*0.628*	−0.194	−0.086	−0.009
	*p*	0.234	0.100	0.087	0.488	^∗∗^ *0.0093*	0.369	0.632	0.828
IET (days)	r	0.061	−0.454	−0.161	0.270	−0.454	−0.039	0.305	0.361
	*p*	0.844	0.147	0.575	0.370	0.128	0.845	0.305	0.278

LVEF: left ventricular ejection fraction; CK-MB: creatine kinase; CET: cardiovascular disease evolution time,; CRP: C-reactive protein; IET: infection evolution time; r: correlation coefficient; *p*: probability value. ^∗^*p* < 0.05, ^∗∗^*p* < 0.01, and^∗∗∗^*p* < 0.001.

**Table 4:** Correlation between Treg cells and different clinical parameters.

Treg cells				
Clinical parameter		% FOXP3 cells	Expression FOXP3 (MFI)	% CD4 + CD25 + FOXP3+ cells
LVEF (%)	r	−0.08	−0.2566	0.2172
	*p*	0.7812	0.3842	0.4716
CK-MB (ng/mL)	r	0.09341	0.03297	0.259
	*p*	0.7647	0.9205	0.39
Troponin-I (ng/mL)	r	−0.0693	−0.02098	0.01404
	*p*	0.8346	0.956	0.9692
C-reactive protein (mg/L)	r	−0.2967	−0.07692	0.2287
	*p*	0.3247	0.8064	0.4493
CET (days)	r	0.233	*0.6989*	−0.1051
	*p*	0.4426	^∗^ *0.0101*	0.5775

LVEF: left ventricular ejection fraction; CK-MB: creatine kinase; CET: cardiovascular disease evolution time; CRP: C-reactive protein; IET: infection evolution time; r: correlation coefficient; p: probability value. ^∗^*p* < 0.05, ^∗∗^*p* < 0.01, and ^∗∗∗^*p* < 0.001.

## Data Availability

The data presented in this study are available on request from the corresponding author. The data are not publicly available because it needed a data-sharing agreement that provides for (1) a commitment to use the data only for research purposes and not to identify any individual participant; (2) a commitment to secure the data using appropriate computer technology; and (3) a commitment to destroy or return the data after analyses are completed.
